# A systematic review of patient safety in mental health: a protocol based on the inpatient setting

**DOI:** 10.1186/s13643-016-0365-7

**Published:** 2016-11-29

**Authors:** Danielle D’Lima, Stephanie Archer, Bethan Ines Thibaut, Sonny Christian Ramtale, Lindsay H. Dewa, Ara Darzi

**Affiliations:** 1 Department of Applied Health Research, 1-19 Torrington Place, London, WC1E 7HB UK; 2 Patient Safety Translational Research Centre, Medical School Building Room 5.03, St Mary’s Campus, Norfolk Place, London, W2 1PG UK; 3 Department of Surgery and Cancer, 10th Floor, Queen Elizabeth the Queen Mother Wing (QEQM), St Mary’s Campus, Norfolk Place, London, W2 1PG UK

**Keywords:** Patient safety, Mental health, Research, Inpatient setting

## Abstract

**Background:**

Despite the growing international interest in patient safety as a discipline, there has been a lack of exploration of its application to mental health. It cannot be assumed that findings based upon physical health in acute care hospitals can be applied to mental health patients, disorders and settings. To the authors’ knowledge, there has only been one review of the literature that focuses on patient safety research in mental health settings, conducted in Canada in 2008. We have identified a need to update this review and develop the methodology in order to strengthen the findings and disseminate internationally for advancement in the field. This systematic review will explore the existing research base on patient safety in mental health within the inpatient setting.

**Methods:**

To conduct this systematic review, a thorough search across multiple databases will be undertaken, based upon four search facets (“mental health”, “patient safety”, “research” and “inpatient setting”). The search strategy has been developed based upon the Canadian review accompanied with input from the National Reporting and Learning System (NRLS) taxonomy of patient safety incidents and the Diagnostic and Statistical Manual of Mental Disorders (fifth edition). The screening process will involve perspectives from at least two researchers at all stages with a third researcher invited to review when discrepancies require resolution. Initial inclusion and exclusion criteria have been developed and will be refined iteratively throughout the process. Quality assessment and data extraction of included articles will be conducted by at least two researchers. A data extraction form will be developed, piloted and iterated as necessary in accordance with the research question. Extracted information will be analysed thematically.

**Discussion:**

We believe that this systematic review will make a significant contribution to the advancement of patient safety in mental health inpatient settings. The findings will enable the development and implementation of interventions to improve the quality of care experienced by patients and support the identification of future research priorities.

**Systematic review registration:**

PROSPERO CRD42016034057

**Electronic supplementary material:**

The online version of this article (doi:10.1186/s13643-016-0365-7) contains supplementary material, which is available to authorized users.

## Background

Patient safety refers to the prevention of harm as a result of receiving healthcare services [1]. This includes events such as mistakes during procedures and accidents that happen during hospital stays. There has been growing interest in patient safety as a discipline in recent years [2, 3]. The vast majority of this body of research to date has focused on physical healthcare; however, it cannot be assumed that findings based upon physical health patients can be applied to mental health patients, disorders and settings. This is due to the different challenges that are presented in this specialised area of care [4].

There has been exploration of specific adverse events that occur within acute mental health facilities, such as suicide and self-harm [5–8], with prevalence rates and precipitating factors examined regularly. There have also been aspects of patient safety touched upon in quality of care research within mental health facilities, for example, audit and quality of care intervention studies [9, 10]. A systematic review has also been conducted collating the literature regarding quality of care for institutionalised mental health patients [11]. This review highlighted incidents such as restraint and seclusion as important aspects of quality of care but did not discuss patient safety as a whole. Although patient safety can be seen as a subset of quality of care, they are not synonymous, with research on the quality of healthcare sometimes lacking a focus on patient safety issues [12].

Research involving patient safety systems and management as a whole is lacking, as are attempts to apply patient safety as a complete concept directly to mental health research [4]. This may be because mental health in general has been viewed as a neglected area in which patients may be less likely to have a voice when it comes to their care and safety [13]. It has also been suggested that the social stigma surrounding mental health issues has the potential in itself to contribute to patient safety being neglected [4].

A Canadian Report published in 2009 [4] is the only review of the literature to our knowledge that has explored the role of patient safety in mental health. This work makes a valid contribution to the emerging field of patient safety in mental health but has flaws that require addressing. Firstly, the literature review is now out of date, with evidence being collected in 2008. Secondly, the work focussed on a pre-specified set of patient safety incidents, so other areas of patient safety concern could have been neglected. Finally, the review was not published in an academic journal as far as we are aware, and there is a lack of methodological detail given in the report, limiting readers' understanding of its methodological rigour. The review does not specify any independent reviewing process, inter-reviewer agreement calculation, inclusion or exclusion criteria or assessment of the quality of the research included. This restricts broader access to the findings and makes it difficult to ensure that the processes of article selection and data extraction were conducted systematically. It is for these reasons that it is thought important to develop the methodology by undertaking a full systematic review as a first step towards a broader research programme on patient safety in mental health at the NIHR Imperial Patient Safety Translational Research Centre (PSTRC). The importance of high-quality systematic reviews in the patient safety research field has been emphasised [14].

This systematic review aims to bring together research within mental health to provide a clear picture of what we know about patient safety within this context. In order to deliver high-quality care to service users, it is essential that a stronger understanding of patient safety in mental health is developed and disseminated.

### Aim

This systematic review aims to report an overview of the existing research base on patient safety in mental health within inpatient settings and critically reflect on the method and quality of existing studies. This review will inform patient safety practices in inpatient mental health settings, highlighting the best practice as well as gaps in knowledge to provide areas for further research that could lead to improvement in patient care.

## Methods

The checklist included as an Additional file 1 has been used to guide the information included in this protocol.

### Eligibility criteria

This systematic review focuses on research conducted within the inpatient setting; this includes two core areas. The first area is mental healthcare inpatient settings (e.g. secure forensic units). The second area is an inpatient setting (i.e. within acute care) where a person with a mental health diagnosis is in receipt of healthcare for a physical problem.

The four key facets for this systematic review are:

- “Mental Health” defined as a field comprising various professions, such as psychiatry and clinical psychology that deal with the promotion of mental and psychological well-being and the prevention, diagnosis or treatment of mental disorders as listed in the Diagnostic and Statistical Manual fifth edition [15]
- “Patient Safety” defined as “The avoidance, prevention and amelioration of adverse outcomes or injuries stemming from the process of healthcare” [1]
- “Research” defined as diligent and systematic inquiry or investigation into a subject in order to produce generalisable knowledge and to discover or revise facts, theories, applications, etc.
- “Inpatient Setting” defined as hospital settings which provide continuous care for a period of over 24 h

The search terms will be restricted to title and abstract only. The time period will be restricted to work published in or after 1999 (in line with the seminal publication of the Institute of Medicine’s report “To Err is Human: Building a Safer Health System” [16]). This review will be purposely broad in order to effectively ground future research in this area.

Initial inclusion and exclusion criteria have been developed based upon the definitions included above and will be further iterated by the research team as part of the ongoing process.

Inclusion criteria:

- Population: Article must report on patients being treated within mental health services (“Mental Health” being defined as a field comprising various professions, such as psychiatry and social work, that deals with the promotion of mental and psychological well-being and the prevention, diagnosis or treatment of mental disorders as listed in the Diagnostic and Statistical Manual fifth edition [15]) or having a diagnosis of mental disorder.
- Intervention/outcomes: Articles must report on interventions or data related to “Patient Safety” (defined as “The avoidance, prevention and amelioration of adverse outcomes or injuries stemming from the process of healthcare” [12]).
- Comparators: There are no restrictions on articles regarding the use or lack of comparison groups
- Timing: Article must be published in, or after, the year 1999.
- Setting: Article must report on the “Inpatient Setting” (defined as hospital settings which provide continuous care for a period of over 24 h).

Exclusion criteria:

- Population: Articles based purely on physical healthcare patients with no connection to mental health and well-being (not mental health)
- Intervention/outcomes: Articles based purely upon non-safety-related issues, e.g. general patient experience or clinical effectiveness of specific treatments (not patient safety)
- Comparators: There are no restrictions on articles regarding the use or lack of comparison groups
- Timing: Articles published before 1999
- Setting: Articles based purely upon primary care, community care or social care (not inpatient setting)

Other general exclusion criteria:

- Articles not in English
- Conference abstracts/protocols/book chapters
- Articles that present opinion/editorials/commentaries/clinical case reviews (not research)

After conducting the title and abstract screening and discussing across the research team, the criteria were developed to specify the focus for the full-text review. The initial additional criteria for the full-text review phase are outlined below:

Exclusion criteria:

- Population: Articles that amalgamate data from both inpatient and outpatient settings such that data for an inpatient only sample is not available
- Interventions/outcomes: Articles that solely examine the reliability or validity of risk assessment tools, with no relation to the management of the risk that the tool is measuring

Other general exclusion criteria:

- Articles that are reviews of any kind (including literature and systematic reviews)
- Articles that are not empirical research (i.e. articles that do not have a clearly defined hypothesis or research question that aims to generate new knowledge in this field of research)

### Study design

We will consider all study designs in this systematic review. Articles must present “research” (defined as diligent and systematic inquiry or investigation into a subject in order to discover or revise facts, theories, applications, etc.)

The core stages of the literature search are listed below. We have currently completed the title and abstract phase. Further detail on each stage is provided later in this protocol:

- Run search and restrict terms to title and abstract only (searches may need to be run individually in each database in order to include the appropriate MeSH terms and specifications etc.)
- Combine searches from all databases and remove duplicates using both an electronic and manual approach
- Title and abstract screening according to inclusion and exclusion criteria
- Full-text screening according to inclusion and exclusion criteria. Hand searching will also be conducted based on reference lists
- Quality and risk of bias assessment
- Data extraction against key research questions
- Synthesis, assessment of the strength of the body of evidence and write up
- Dissemination (i.e. publication and presentation) and application (i.e. through the development of research studies)

**Fig. 1:**
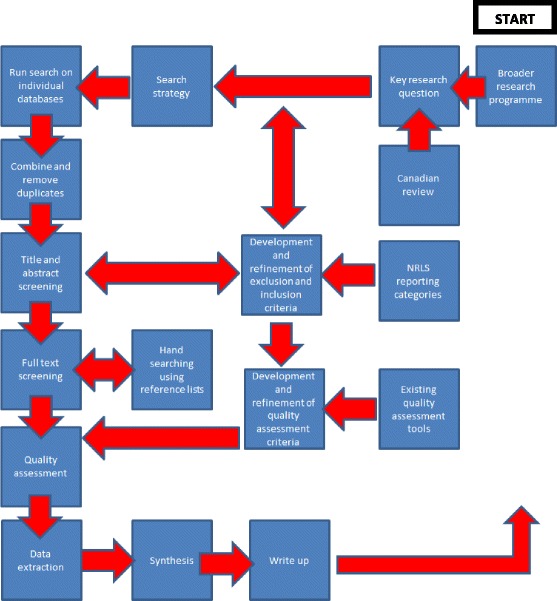
Flow diagram demonstrating how the stages of the systematic review are expected to interact with and influence one another

However, it is important to emphasise that in reality, a systematic review is not usually a simple linear process but instead is iterative in nature [14]. The flow diagram included below in Fig. 1 demonstrates how the stages are expected to interact with and influence one another. Further iterations of this flow chart will be developed throughout the project to represent changes and to capture key numbers. This protocol will also be updated and amended as and when necessary throughout the review process.

### Search strategy

Our search strategy is presented below in Table 1. The taxonomy used in the NRLS for England and Wales has been used to identify additional search terms associated with patient safety that were not included in the original Canadian review due to their restrictions on included incident types. The taxonomy was not amended, to ensure all incident types that may be included in the literature were picked up in our search and to avoid any assumptions being made by the researchers about the relative importance of incident types within mental health. The search terms have been developed by gaining multiple expert perspectives on various iterations of them. They have been iterated to include the appropriate truncation and syntax in order to run the searches on each individual database of interest.

**Table 1:** Search terms against each facet

Mental Health	Patient Safety	Research	Inpatient Setting
Mental health.ti,ab. Mental wellbeing.ti,ab. Mental well being.ti,ab. Psychological well being.ti,ab. Psychological wellbeing.ti,ab. Mental disorder*1.ti,ab Mental illness*2.ti,ab. Mental disease*1.ti,ab. Psychiatr*.ti,ab. Anxiety disorder*1.ti,ab. Delirium.ti,ab. Dementia.ti,ab. Dissociative disorder*1.ti,ab. Factitious disorder*1.ti,ab. Impulse control disorder*1.ti,ab. Mood disorder*1.ti,ab. Affective disorder*1.ti,ab. Psychotic disorder*1.ti,ab. Depressive disorder*1.ti,ab. Neurotic disorder*1.ti,ab. Personality disorder*1.ti,ab. Conduct disorder*1.ti,ab. Schizophreni*.ti,ab. Somatoform disorder*1.ti,ab. Substance related disorder*1.ti,ab. Clinical Psychology.ti,ab. Impulsive behavio?r.ti,ab. Adjustment disorder*1.ti,ab. Eating disorder*1.ti,ab. Sleep disorder*1.ti,ab. Neuros#s.ti,ab. Psychos#s.ti,ab. Delusion*.ti,ab. Paranoia.ti,ab. Hallucination*1.ti,ab. Addiction*1.ti,ab. Dependence.ti,ab. Misuse.ti,ab. New psychoactive substance*1.ti,ab. Legal high*1.ti,ab. Depression.ti,ab. Panic disorder*1.ti,ab. Phobia*1.ti,ab. Health anxiet*.ti,ab. Bipolar disorder*1.ti,ab. Alcohol abuse.ti,ab. Alcoholism.ti,ab. Obsessive compulsive disorder*1.ti,ab. Obsessive thought*1.ti,ab. Intrusive thought*1.ti,ab. Post traumatic stress disorder*1.ti,ab. Post-traumatic stress disorder*1.ti,ab. Cognitive Behavio?ral Therap*.ti,ab. Psychotherap*.ti,ab. Person centred therap*.ti,ab. Person-centred therap*.ti,ab. Counselling.ti,ab. Antidepressant medication*1.ti,ab. Antipsychotic medication*1.ti,ab. Antianxiety medication*1.ti,ab. Psychotropic medication*1.ti,ab. Mindfulness based cognitive therap*.ti,ab. Mindfulness-based cognitive therap*.ti,ab. Mindfulness based relapse prevention.ti,ab. Mindfulness-based relapse prevention.ti,ab. Mindfulness based stress reduction.ti,ab. Mindfulness-based stress reduction.ti,ab. Electroconvulsive therap*.ti,ab. Verbal deescalation.ti,ab. Therapeutic.ti,ab. Functional Analys#s.ti,ab. Dialectical Behavio?r Therap*.ti,ab. Dysexecutive syndrome.ti,ab.	Patient safety.ti,ab. Adverse event*1.ti,ab. Adverse drug event*1.ti,ab. Sentinel event*1.ti,ab. Incident*1.ti,ab. Error*1.ti,ab. Near miss*2.ti,ab. Close call*1.ti,ab. Never event*1.ti,ab. Critical outcome*1.ti,ab. Adverse outcome*1.ti,ab. Unanticipated outcome*1.ti,ab. Suicide*1.ti,ab. Self-harm.ti,ab. Self harm.ti,ab. Behavio?r control.ti,ab. Restraint.ti,ab. Seclusion.ti,ab. Safety management.ti,ab. Failure to diagnose.ti,ab. Failure of diagnos#s.ti,ab. Under diagnosis.ti,ab. Over diagnosis.ti,ab. Misdiagnosis.ti,ab. Dual diagnos#s.ti,ab. Delay in diagnos#s.ti,ab. Wrong diagnos#s.ti,ab. Incorrect diagnos#s.ti,ab. Safety culture.ti,ab. Safety climate.ti,ab. Fall*1.ti,ab. Slip*1.ti,ab. Trip*1.ti,ab. Falling.ti,ab. Slipping.ti,ab. Tripping.ti,ab. Accident prevention.ti,ab. Patient accident*1.ti,ab. Patient in road traffic accident*1.ti,ab. Collision with an object.ti,ab. Contact with an object.ti,ab. Contact with sharp*1.ti,ab. Collision with sharp*1.ti,ab. Exposure to hazardous substance*1.ti,ab. Inappropriate patient handling.ti,ab. Inappropriate patient positioning.ti,ab. Elope.ti,ab. Wander.ti,ab. Runaway.ti,ab. Abscond*.ti,ab. Escorted leave.ti,ab. Unescorted leave.ti,ab. Aggressi*.ti,ab. Violence.ti,ab. Assault*1.ti,ab. Abus*.ti,ab. Disruptive behavio?r.ti,ab. Racial attack*1.ti,ab. Sexual attack*1.ti,ab. Sexually inappropriate.ti,ab. Physical attack*1.ti,ab. Verbal attack*1.ti,ab. Missing patient*1.ti,ab. Failure in access.ti,ab. Unexpected readmission*1.ti,ab. Reattendance*1.ti,ab. Unplanned admission*1.ti,ab. Transfer to specialist care unit*1.ti,ab. Delay in discharge.ti,ab. Failure to discharge.ti,ab. Inappropriate discharge.ti,ab. Planning failure.ti,ab. Self discharge.ti,ab. Self-discharge.ti,ab. Discharge against medical advice.ti,ab. Failure in referral process*.ti,ab. Failure to return from authorised leave.ti,ab. Transfer delay*1.ti,ab. Transfer failure*1.ti,ab. Inappropriate transfer*1.ti,ab. Unsafe transfer*1.ti,ab. Unsafe clinical environment*1.ti,ab. Inappropriate clinical environment*1.ti,ab. Inappropriate admission of a minor to an adult setting.ti,ab. Inappropriate transfer of a minor to an adult setting.ti,ab. Poor clinical assessment*1.ti,ab. Lack of clinical assessment*1.ti,ab. Lack of risk assessment*1.ti,ab. Wrong scan*1.ti,ab. Wrong x-ray*1.ti,ab. Wrong specimen*1.ti,ab. Inadequate scan*1.ti,ab. Inadequate x-ray*1.ti,ab. Inadequate specimen*1.ti,ab. Incomplete scan*1.ti,ab. Incomplete x-ray*1.ti,ab. Incomplete specimen*1.ti,ab. Mislabelled scan*1.ti,ab. Mislabelled x-ray*1.ti,ab. Mislabelled specimen*1.ti,ab. Unlabelled scan*1.ti,ab. Unlabelled x-ray*1.ti,ab. Unlabelled specimen*1.ti,ab. Missing scan*1.ti,ab. Missing x-ray*1.ti,ab. Missing specimen*1.ti,ab. Failure to interpret test result*1.ti,ab. Delay to interpret test result*1.ti,ab. Failure to act on test result*1.ti,ab. Delay to act on test result*1.ti,ab. Failure to receive test result*1.ti,ab. Delay to receive test result*1.ti,ab. Incorrect test result*1.ti,ab. Incorrect report*1.ti,ab. Missing test result*1.ti,ab. Missing report*1.ti,ab. Failure to undertake test*.ti,ab. Delay to undertake test*.ti,ab. Patient confidentiality.ti,ab. Communication failure*1.ti,ab. Failed communication*1.ti,ab. Failure in communication*1.ti,ab. Failure to receive informed consent.ti,ab. Inadequate handover.ti,ab. Documentation delay*1.ti,ab. Mislabelled documentation.ti,ab. Missing documentation.ti,ab. Inadequate documentation.ti,ab. Wrong documentation.ti,ab. Illegible documentation.ti,ab. Mislabelled healthcare record*1.ti,ab. Inadequate healthcare record*1.ti,ab. Missing healthcare record*1.ti,ab. Wrong healthcare record*1.ti,ab. Illegible healthcare record*1.ti,ab. Mislabelled referral letter*1.ti,ab. Inadequate referral letter*1.ti,ab. Missing referral letter*1.ti,ab. Wrong referral letter*1.ti,ab. Illegible referral letter*1.ti,ab. Misfiled documentation.ti,ab. No access to documentation.ti,ab. Patient incorrectly identified.ti,ab. Delay in obtaining clinical assistance.ti,ab. Difficulty in obtaining clinical assistance.ti,ab. Delay in recogni#ing complication*1 of treatment.ti,ab. Failure in recogni#ing complication*1 of treatment.ti,ab. Delay in monitoring.ti,ab. Failure to monitor.ti,ab. Failure to follow up.ti,ab. Infection Control.ti,ab. Failure of sterili#ation of equipment.ti,ab. Contamination of equipment.ti,ab. Health care acquired infection*1.ti,ab. Healthcare acquired infection*1.ti,ab. Health care associated infection*1.ti,ab. Healthcare associated infection*1.ti,ab. Wound infection*1.ti,ab. Surgical site infection*1.ti,ab. Unsafe environment*1.ti,ab. Inappropriate environment*1.ti,ab. Unsafe equipment.ti,ab. Inappropriate equipment.ti,ab. Availability of equipment.ti,ab. Availability of bed*1.ti,ab. Availability of IT.ti,ab. Staff shortage*1.ti,ab. Unavailability of staff.ti,ab. Lack of skilled staff.ti,ab. Unskilled staff.ti,ab. Lack of suitably trained staff.ti,ab. Failure of device*1.ti,ab. Failure of equipment.ti,ab. Unavailability of device*1.ti,ab. Extended stay.ti,ab. Extended episode*1 of care.ti,ab. Failure to discontinue treatment*1.ti,ab. Infusion injur*.ti,ab. Missing needle*1.ti,ab. Missing swab*1.ti,ab. Missing instrument*1.ti,ab. Retained needle*1.ti,ab. Retained swab*1.ti,ab. Retained instrument*1.ti,ab. Theatre list details incorrect.ti,ab. Inappropriate treatment*1.ti,ab. Wrong treatment*1.ti,ab. Unplanned return to theatre.ti,ab. Maternal death*1.ti,ab. Anaesthetic complication*1.ti,ab. Intensive Therapy Unit Admission*1.ti,ab. Intensive Treatment Unit Admission*1.ti,ab. Intensive Care Unit Admission*1.ti,ab. Venous thromboembolism*1.ti,ab. Pulmonary embolism*1.ti,ab. Readmission of mother.ti,ab. Stillbirth*1.ti,ab. Neonatal death*1.ti,ab. Birth trauma*1.ti,ab. Term baby admitted to neonatal unit.ti,ab. Undiagnosed f?etal abnormalit*.ti,ab. Pressure ulcer*1.ti,ab. Padded room*1.ti,ab. Ligature point*1.ti,ab. Self-neglect.ti,ab. Self neglect.ti,ab. Splint*1.ti,ab. Head bang*.ti,ab. Head-bang*.ti,ab.	Research.ti,ab. Academic work.ti,ab. Academic understanding.ti,ab. Theor*.ti,ab. Randomised controlled trial*1.ti,ab. Controlled clinical trial*1.ti,ab. Random allocation.ti,ab. Double blind method.ti,ab. Single blind method.ti,ab. Single blind stud*.ti,ab. Double blind stud*.ti,ab. Triple blind stud*.ti,ab. Multicentre stud*.ti,ab. Random sample*1.ti,ab. Evidence base*1.ti,ab. Evidence scan*1.ti,ab. Systematic review*1.ti,ab. Scoping review*1.ti,ab. Narrative review*1.ti,ab. Literature review*1.ti,ab. Meta narrative*1.ti,ab. Meta synthesi*.ti,ab. Meta-analys*.ti,ab. Clinical trial*1.ti,ab. Placebo*1.ti,ab. Comparative stud*.ti,ab. Evaluation stud*.ti,ab. Evaluative stud*.ti,ab. Descriptive stud*.ti,ab. Community trial*1.ti,ab. Follow up stud*.ti,ab. Prospective stud*.ti,ab. Longitudinal stud*.ti,ab. Qualitative.ti,ab. Quantitative.ti,ab. Focus group*1.ti,ab. Semi-structured interview*1.ti,ab. Quality improvement project*1.ti,ab. Data collection.ti,ab. Data analysis.ti,ab. Survey*1.ti,ab. Observation*1.ti,ab. Ethnograph*.ti,ab. Intervention*1.ti,ab. Investigation*1.ti,ab. Experiment*.ti,ab. Case stud*.ti,ab. Delphi.ti,ab. Nominal group technique*1.ti,ab. Nominal group stud*.ti,ab. Consensus stud*.ti,ab.	Hospital*.ti,ab. Acute care.ti,ab. Secondary care.ti,ab. Tertiary care.ti,ab. Unit*1.ti,ab. Ward*1.ti,ab. Low secure.ti,ab. Medium secure.ti,ab. High secure.ti,ab. Secure facilit*.ti,ab. Forensic*1.ti,ab. Inpatient*1.ti,ab. Triage.ti,ab.

### Databases

- MEDLINE
- Embase
- Health Management Information Consortium (HMIC)
- PsychInfo
- Web of Science
- Cumulative Index to Nursing and Allied Health Literature (CINAHL)

### Grey literature and hand searching

Google and Google Scholar will also be searched using key phrases in line with the search strategy. The first 20 pages of results will be screened initially, as a large number of results are anticipated, and time resources need to be considered. The amount of new information being garnered from these first 20 pages will be assessed, and the page limit extended if it is felt that this would produce more original data. The reference lists of the eligible articles identified after full-text screening by the main search strategy will be reviewed by the research team to ensure that no key articles have been missed.

### Screening

Perspectives from at least two reviewers will be gained on decision-making throughout the screening process. Pilot screening of a random sample of abstracts has been conducted with at least two reviewers screening independently. Disagreements have been discussed with other researchers within the team to gain consensus and to adapt and clarify the inclusion and exclusion criteria if needed.

After this stage, one reviewer has screened all abstracts, with a second reviewer independently screening a random sample of 10%. A kappa statistic will be calculated to assess inter-reviewer agreement. Full text screening is currently underway with input being provided from all members of the research team.

### Critical appraisal

Studies will not be excluded on the basis of quality, but the quality of studies will be assessed and presented descriptively in order to inform the current state of knowledge in this area. Hawker et al.’s [17] quality assessment tool will be used to appraise studies, as the data garnered through this systematic review is anticipated to be varied and this tool is designed to be applicable to both quantitative and qualitative studies. Hawker’s checklist evaluates nine domains: (1) abstract/title, (2) introduction and aims, (3) method and data, (4) sampling, (5) data analysis, (6) ethics and bias, (7) results, (8) transferability and generalisability and (9) implications and usefulness. The quality of these nine domains will be assessed according to four descriptors: very poor, poor, fair and good. A global assessment of the quality of each article will then be undertaken by collating this information. This will allow for evaluation across the variety of methodologies we anticipate to garner from this review. The quality assessment tool will be applied to all included articles by two reviewers, and results will be compared and discussed with input from the broader research team.

### Data extraction

Data will be extracted from included articles in line with the core research question driving this systematic review. A data extraction form will be developed, piloted and applied across all articles. If key information is not available in an article, the research team will contact the authors and request additional data. Again, perspectives from at least two reviewers will be employed to ensure that the relevant data is selected and extracted in the most systematic way possible. Table 2 is an example of the type of evidence table that will be used to record and extract data from included articles.

**Table 2:** Sample evidence table

Author, year	Study design	Setting	Participants	Aims/objectives	Outcomes/data related to patient safety	Study quality
Mezey, Hassell & Batlett (2005)	Qualitative Interview	Medium-secure NHS psychiatric units (England and Wales)	31 female inpatients 58 staff (9 consultant forensic psychiatrists, 9 ward managers, 18 staff nurses, 9 nursing support workers, 3 social workers, 5 psychologists and 5 occupational therapist). 53% women, 47% men.	To examine the impact of gender segregation on the safety of women patients detained in medium-secure psychiatric facilities.	Women patients in both types of units reported high levels of actual and threatened physical and sexual violence. Women in single-sex units reported intimidation, threats and abuse by other women patients, although they were less vulnerable to sexual abuse and exploitation and serious physical assault.	Will be assessed and scored from ‘Good’ to ‘Very Poor’ based upon Hawker et al. (2002)
Meehan, Morrison & McDougall (1999)	Mixed methods Case review and interview	Acute psychiatric unit on the grounds of a public hospital	Case review: All participants who were recorded as AWOL in the unit’s register within a 6-month study period. Interviews: 14 patients (9 males, 5 females; 19–58 years old) who were interviewed within 48 h of returning from being AWOL.	To identify patient and environmental characteristics associated with absconding behaviour and to gain an understanding of the behaviour form the patient’s perspective.	Those who absconded were male (58%), under 40 years of age (74%), admitted involuntarily (78%), and had a diagnosis of schizophrenia (42%). One third of all AWOL incidents resulted from repeated absconding by the same individuals. The first 7 days post admission was a high-risk period for absconding behaviour. Identified situational and environmental factors likely to increase the risk of absconding included: staff skills, communication and management strategies.	

### Bibliographic management

Software such as Endnote and Excel are being used to keep track of references and decision-making throughout the process. Endnote is being used to store and group references. Excel spreadsheets are being used to apply screening criteria systematically, record the reasoning behind decision-making and extract data from high-relevance articles in line with the research question.

### Data synthesis

Data synthesis is likely to be thematic in nature and influenced by the core research question (i.e. identifying key findings from the existing research base that can help set a future research agenda).

We will use a narrative synthesis approach as the data retrieved is likely to be heterogeneous, conducting meta-analysis/synthesis if and where appropriate.

### Write-up, dissemination and use

The results will be written up in accordance with the PRISMA guidelines for peer-reviewed publication to support existing understanding and future research in this important area. They will also be used locally to support the development of a programme of work to improve patient safety in mental health in collaboration with local trusts.

## Discussion

### Strengths and limitations of the review

The main strength of this systematic review is that it will be purposely broad in terms of patient safety issues and is expected to cover a wide range of literature. This will ensure that the review captures all relevant data and we are able to build a complete picture of the current state of the research area. The limitations will be that it will exclude articles that were published before 1999 and those that are not written in English and therefore may run the risk of bias, by presenting an ethnocentric view of the evidence. The focus on the inpatient setting alone may also be considered a criticism of this review, as we have therefore had to exclude any research conducted in other mental health settings that may have offered different research perspectives. However, we believe that different patient safety issues are likely to occur in different settings (i.e. inpatient setting compared to primary, community and social care) and therefore, it is important to explore them separately. We have chosen to focus on the inpatient setting as a priority due to its relation to our initial work programme as a translational patient safety research centre. Systematic reviews focussing upon primary, community and social care form part of our future work plan.

### Relevance of the review

The findings of this systematic review will support the development of future academic research programmes based upon patient safety in mental health. They will also support the design of interventions to protect patient safety in this area and improve the quality of care that individuals receive. In this sense, they will be of relevance to multiple groups including patients, researchers, policy makers, healthcare managers and clinicians.

## Additional file

Additional file 1: PRISMA-P (Preferred Reporting Items for Systematic review and Meta-Analysis Protocols) 2015 checklist: recommended items to address in a systematic review protocol*. (DOC 81 kb)
